# Development and utilization of an open-data, web-based geographic information system to support the response to the 2024 Noto Peninsula earthquake, Japan

**DOI:** 10.5365/wpsar.2026.17.1.1277

**Published:** 2019-03-16

**Authors:** Ryo Horiike, Tomoya Itatani, Hisao Nakai, Kentaro Tanaka

**Affiliations:** a School of Medicine, Department of Nursing, Nara Medical University, Nara, Japan.; b Division of Home Care Nursing, Department of Fundamental and Community Nursing Science, School of Nursing, Faculty of Medicine, University of Miyazaki, Miyazaki, Japan.; c Faculty of Nursing, University of Kochi, Kochi, Japan.; d Graduate School of Nursing, Department of Nursing, Osaka Metropolitan University, Osaka, Japan.

On 1 January 2024, an earthquake of magnitude 7.6 struck the Noto Peninsula in Japan, causing over 500 deaths and damaging about 160 000 houses. (*1*) Eight months later, torrential rains in the same area caused a further 14 fatalities and extensive flooding. (*2*) During the first 4 months of the response, 15 489 people, including public health nurses (PHNs) and Disaster Health Emergency Assistance Teams, were dispatched to Ishikawa Prefecture. (*3*) Aggregated, real-time disaster data are available to support disaster response via the Shared Information Platform for Disaster Management and the Disaster Digital Information System for Health and Well-being, but they are rarely accessible to front-line personnel before their deployment to disaster-affected areas. (*4*) In early January 2024, we developed an open-data Public Health Nursing map (PHN-Map) on Web-based Geographic Information Systems (WebGIS) architecture, to support public health nursing activities by providing up-to-date situational information and training resources before deployment to the Noto Peninsula. This report describes the development of the WebGIS system and the subsequent addition of 360° images to PHN-Map in September 2024, as well as the results of a user survey.

## Methods

### Data integration

Between 2 and 7 January 2024, we downloaded data from National Land Numerical Information, a database of the Geospatial Information Authority of Japan. The extracted data included administrative areas, emergency transport roads, and medical and public facilities. (*5*) Additional data sets, namely 2020 census grid statistics (the 250-m population mesh from the Statistics Bureau via e-Stat portal (*6*)) and municipal open data on evacuees, isolated hamlets and temporary housing, were downloaded from public web sites (**Table 1**). Data sets were used to create thematic layers including administrative boundaries, emergency transport roads, medical facilities, shelters, evacuee counts, populations of isolated hamlets and temporary housing.

**Table 1:** Layers of the PHN-Map

No.	Data publication period	Type of disaster	Data name	Source	URL
1	Post-disaster	Earthquake and torrential rain	Number of people in isolated settlements	Ishikawa Prefecture Disaster Response headquarters meeting materials	https://www.pref.ishikawa.lg.jp/saigai/202401jishin-taisakuhonbu.html#honbu
2	Post-disaster	Earthquake and torrential rain	Number of evacuees in shelters (Nanao City)	raokiey and Nanao City web site	https://github.com/raokiey/R06-Noto-Peninsula-EQ-open-shelter-Nanao/blob/main/README.md
3	Post-disaster	Earthquake and torrential rain	Number of emergency temporary housing	MASAMURA Akinobu, Tokyo Metropolitan University	https://github.com/a-masumura/R06-Noto-Peninsula-EQ-temporary-housing/
4	Post-disaster	Earthquake	DHEAT, number of dispatched public health nurses	Ministry of Health, Labour and Welfare web site	https://www.mhlw.go.jp/stf/newpage_37198.html
5	Post-disaster	Earthquake and torrential rain	Landslide and deposition distribution	Geospatial Information Authority of Japan	https://www.gsi.go.jp/BOUSAI/20240101_noto_earthquake.html#6-1
6	Post-disaster	Earthquake	Tsunami inundation area	Geospatial Information Authority of Japan	https://www.gsi.go.jp/BOUSAI/20240101_noto_earthquake.html#7
7	Post-disaster	Earthquake and torrential rain	Inundation area by heavy rainfall	National Research Institute for Earth Science and Disaster Resilience	https://mizu.bosai.go.jp/wiki2/wiki.cgi?page=%CE%E1%CF%C26%C7%AF9%B7%EE21%C6%FC%A4%AB%A4%E9%A4%CE%C2%E7%B1%AB
8	Post-disaster	Earthquake	Open building footprints	Google	https://data.humdata.org/dataset/open_buildings_v3_west_japan_earthquake_epicenter? (English)
9	Post-disaster	Earthquake and torrential rain	Aerial photographs (orthoimages)	GSI tiles (Geospatial Information Authority of Japan)	https://maps.gsi.go.jp/development/ichiran.html#t20240102noto_suzu_0114do
10	Post-disaster	Earthquake and torrential rain	Emergency restored road sections	Ministry of Land, Infrastructure, Transport and Tourism road restoration visualization map	https://www.mlit.go.jp/road/r6noto/index2.html (Japanese and English)
11	Post-disaster	Earthquake and torrential rain	Damaged road locations	Ministry of Land, Infrastructure, Transport and Tourism road restoration visualization map	https://www.mlit.go.jp/road/r6noto/index2.html (Japanese and English)
12	Post-disaster	Earthquake	CS stereographic map	Forestry and Forest Products Research Institute, National Research and Development Agency	https://www2.ffpri.go.jp/soilmap/data-src.html
13	Post-disaster	Earthquake and torrential rain	360° imagery	First author	https://gisphn.github.io/360-image-viewer/
14	Normal times	ND	Administrative districts	National Land Numerical Information, Japan	https://nlftp.mlit.go.jp/ksj/gml/datalist/KsjTmplt-N03-v3_1.html
15	Normal times	ND	Peninsula circulatory roads	Digital National Land Information	https://nlftp.mlit.go.jp/ksj/gml/datalist/KsjTmplt-A37.html
16	Normal times	ND	Sediment disaster alert areas	Digital National Land Information	https://nlftp.mlit.go.jp/ksj/gml/datalist/KsjTmplt-A33-v2_0.html
17	Normal times	ND	Tsunami inundation anticipation areas	Digital National Land Information	https://nlftp.mlit.go.jp/ksj/gml/datalist/KsjTmplt-A40-v2_1.html
18	Normal times	ND	Schools	Digital National Land Information	https://nlftp.mlit.go.jp/ksj/gml/datalist/KsjTmplt-P29-v2_0.html
19	Normal times	ND	Medical facilities	Digital National Land Information	https://nlftp.mlit.go.jp/ksj/gml/datalist/KsjTmplt-P04-v3_0.html
20	Normal times	ND	Emergency transport roads	Digital National Land Information	https://nlftp.mlit.go.jp/ksj/gml/datalist/KsjTmplt-N10-v2_0.html
21	Normal times	ND	National and prefectural agencies	Digital National Land Information	https://nlftp.mlit.go.jp/ksj/gml/datalist/KsjTmplt-P28-v2_0.html
22	Normal times	ND	Municipal offices and public assembly facilities	Digital National Land Information	https://nlftp.mlit.go.jp/ksj/gml/datalist/KsjTmplt-P05-v3_0.html
23	Normal times	ND	Population (250-m mesh)	The Portal Site of Official Statistics of Japan (e-Stat)	https://www.e-stat.go.jp/gis/statmap-search?type=1 (Japanese and English)
24	Normal times	ND	Disaster base hospitals	Ishikawa Prefecture Open Data Catalogue	https://ckan.opendata.pref.ishikawa.lg.jp/dataset/170003_saigai_hospital
25	Normal times	ND	Public facilities	Ishikawa Prefecture Open Data Catalogue	https://ckan.opendata.pref.ishikawa.lg.jp/dataset/170003_public_facility
26	Normal times	ND	Designated shelters	Ishikawa Prefecture Open Data Catalogue	https://ckan.opendata.pref.ishikawa.lg.jp/dataset/170003_evacuation_space

ND: not determined.

Several layers required trimming or reformatting before being added to the WebGIS platform and were edited in the Quantum Geographic Information System (QGIS). (*7*) To maximize speed of deployment, we used Felt, (*8*) a cloud-based collaborative mapping platform that connects spatial data sets and allows users to build interactive maps accessible from any device, including laptops used by PHNs in the field. The system also enables users to view their own location while exploring the map, facilitating situational awareness during disaster response. The use of WebGIS and the Felt software facilitated the integration of heterogeneous data sets into one platform, making it possible to visualize the distribution of evacuation centres, hazard areas and the population in a single image. The map was released publicly on 8 January 2024. After the torrential rains on 20–23 September 2024, inundation and landslide polygons plus new shelters were added; currently, 26 thematic layers are updated as needed.

### 360° imagery

In March 2024 and March 2025 (2 and 14 months post-quake, respectively), Insta360 X3 cameras recorded 360° photos at key sites in the Noto Peninsula. Images were published through an open-source viewer. (*9*) The URL for each photo was linked to its coordinates in PHN-Map pop-ups, enabling users to view the images in either a web browser or a head-mounted display, while 360° imagery provided spatial context beyond the 2D maps, enabling PHNs to virtually assess disaster conditions. Precautions included shooting when only a few people were present, avoiding non-researcher faces, and obscuring vehicle licence plates.

### User feedback

With support from the National Association of Chief Public Health Nurses, an online questionnaire comprising three 10-point Likert-type questions was e-mailed to 5549 PHNs nationwide. The questionnaire was open from 11 April to 14 June 2024. It contained three questions:

1. How easy was PHN-Map to use?
2. How useful was PHN-Map for your work during the Noto Peninsula earthquake?
3. How necessary will PHN-Map be for future disaster PHN activities?

Respondents could also add free-text comments.

## Results

**Fig. 1:**
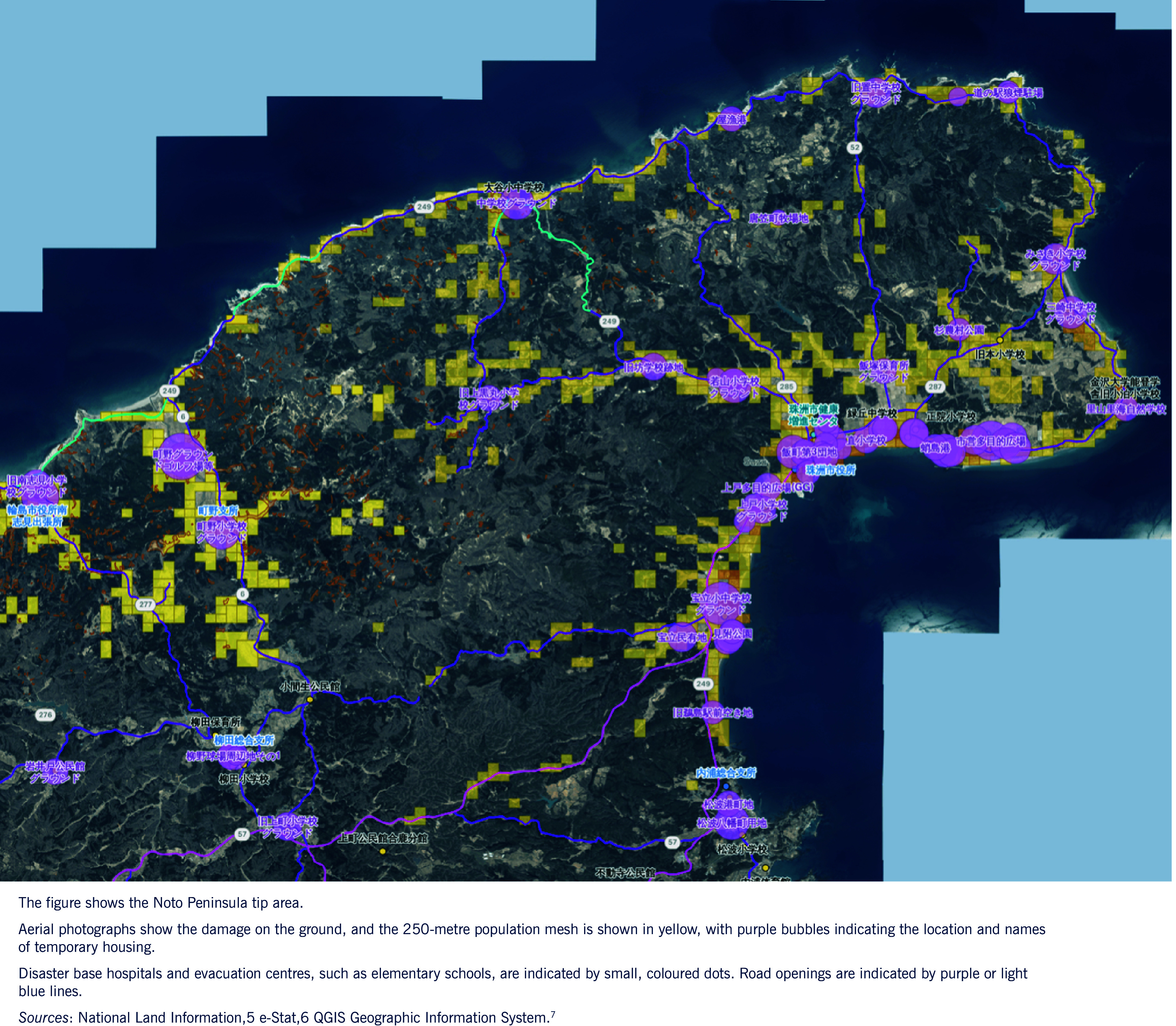
Public health nursing activity-support WebGIS (PHN-Map), as of January 2025

Analytics recorded 301 visits on launch day (8 January 2024) and a weekday mean of 84 visits from 9 to 30 January 2024. After flood layers were added, visits peaked at 212 on 24 September 2024. **Fig. 1** shows the PHN-Map of the Noto Peninsula that was made publicly available on 8 January 2024. The figure presents the version updated as of January 2025, incorporating additional data sets accumulated since the initial release. The map was generated from government aerial imagery and OpenStreetMap tiles and overlaid with symbols indicating damage status, shelters, PHN team bases, populated areas and temporary housing.

[**insert** Figure 1]

**Fig. 2:**
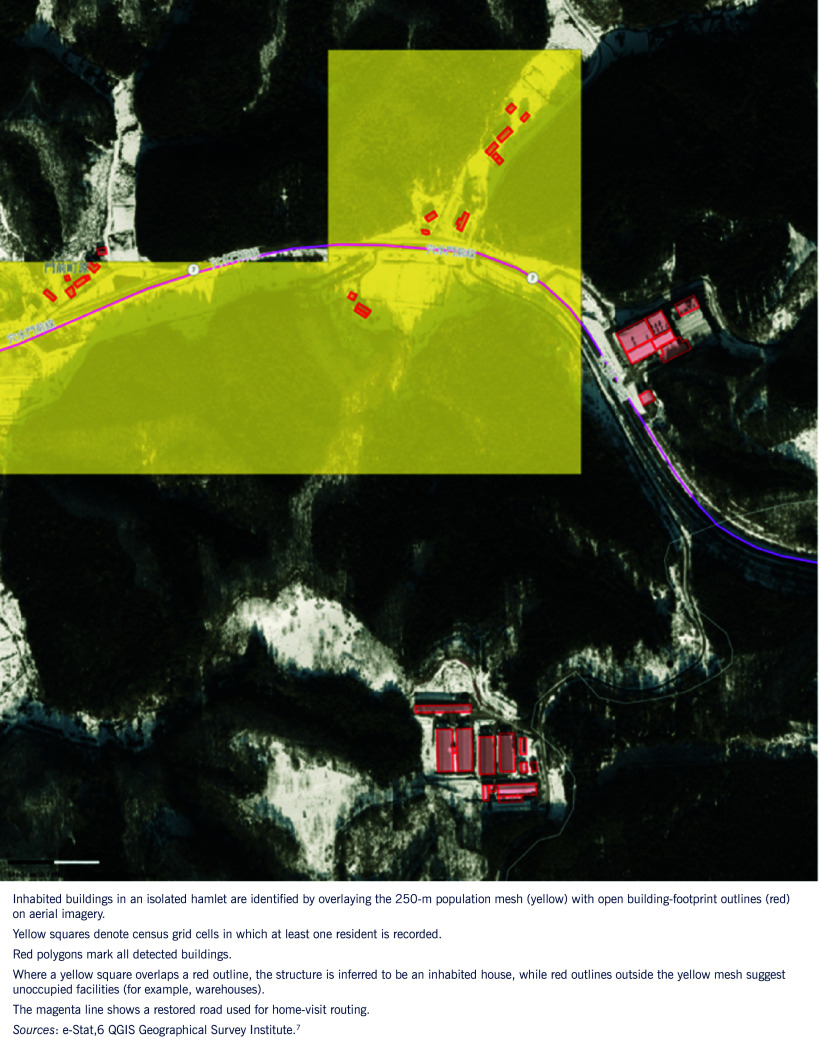
Home-visit routing

Reported field uses, derived from both web site analytics and survey comments, included:

- pre-deployment triage: matching shelter density and road status to PHN specialties;
- flood-risk reassessment: overlapping flood polygons and earthquake damage zones to anticipate waterborne risks;
- home visit routing: identifying isolated households by intersecting population and building footprint data (**Fig. 2**); and
- training and debriefing: using 360° imagery to simulate field conditions for non-deployed PHNs.

The PHN-Map was created by the authors as a ready-made interactive WebGIS integrating multiple data layers; however, users could freely select which layers to display depending on their needs.

[**insert** Figure 2]

### User survey

The questionnaire received 48 responses (response rate: 0.9%). (*10*) Survey results were: usability 5.5/10 (95% CI: 5.1–6.0), usefulness for earthquake response 5.5/10 (4.9–6.1) and need for future disasters 7.7/10 (7.2–8.3). PHNs who were inexperienced with GIS valued future need as highly as experienced peers. Free-text comments included requests for tutorials and lighter data formats.

## Discussion

PHN-Map, constructed entirely from open data, reduced the “land-knowledge gap” among outside responders deployed to the Noto Peninsula earthquake, aligning with prior evidence that rapid GIS visualization can improve the efficiency of disaster resource allocation. (*11*) Integrating 360° media also provided an engaging educational tool for pre-service PHN students and disaster-naïve PHNs, who could virtually experience the affected area and rehearse response scenarios with high immersion. (*9*)

The main system development challenges included the need for manual geocoding of municipal PDF reports without coordinates; this work is labour-intensive, time-consuming and risks misplacing features. Large file sizes were also identified by both developers during implementation and users in survey responses, as a technical limitation that slowed transfers on low-bandwidth disaster networks and could exceed laptop capacity. Ongoing work includes developing automated PDF parsing and vector-tile conversion to address these issues. The survey’s very low response rate likely reflected fatigue among PHNs engaged in disaster response and the use of a broad mailing list, making invitations easy to overlook. This represents a limitation in the evaluation process rather than of the system itself. Further studies are needed to evaluate the system’s effectiveness in disaster exercises, assess PHN training needs, and explore the integration of real-time or crowd-sourced data.

### Conclusion

An open-source WebGIS enriched with 360° imagery furnished PHNs with actionable, up-to-date information during a dual disaster of earthquake and floods. The system also addressed previous problems by enabling pre-deployment overview, the integration of multiagency data, and improved spatial understanding via 360° imagery, which also served as training material for non-deployed PHNs. Routine GIS use in everyday PHN work is recommended, as everyday use will increase PHNs’ and other responders’ proficiency with GIS tools when a disaster occurs.
